# Uranium in phosphate rocks and mineral fertilizers applied to agricultural soils in East Africa

**DOI:** 10.1007/s11356-022-24574-5

**Published:** 2022-12-11

**Authors:** Dennis A. Mwalongo, Nils H. Haneklaus, Jacob B. Lisuma, Thomas T. Kivevele, Kelvin M. Mtei

**Affiliations:** 1 Tanzania Atomic Energy Commission, Directorate of Nuclear Technology and Technical Services, P. O. Box 743, Arusha, Tanzania; 2 Technische Universität Bergakademie Freiberg, Institute of Technical Chemistry, Leipziger Straße 29, Freiberg, Germany; 3 Nelson Mandela African Institution of Science and Technology (NM-AIST), School for Materials, Energy, Water, Environmental Science and Engineering, P.O. Box 447, Arusha, Tanzania; 4 Universität für Weiterbildung Krems, Td Lab Sustainable Mineral Resources, Dr.-Karl-Dorrek-Straße 30, 3500 Krems an Der Donau, Austria; 5 Tobacco Research Institute of Tanzania (TORITA), P.O. Box 431, Tabora, Tanzania

**Keywords:** Phosphate rock, Mineral fertilizer, Uranium, East Africa, Environmental pollution

## Abstract

Phosphate rock, pre-concentrated phosphate ore, is the primary raw material for the production of mineral phosphate fertilizer. Phosphate rock is among the fifth most mined materials on earth, and it is also mined and processed to fertilizers in East Africa. Phosphate ore can contain relevant heavy metal impurities such as toxic cadmium and radiotoxic uranium. Prolonged use of phosphate rock powder as a fertilizer and application of mineral fertilizers derived from phosphate rock on agricultural soils can lead to an accumulation of heavy metals that can then pose an environmental risk. This work assesses the uranium concentrations in four major phosphate rocks originating from East Africa and four mineral phosphate fertilizers commonly used in the region. The concentration measurements were performed using energy-dispersive X-ray fluorescence spectrometry. The results showed that the uranium concentration in phosphate rock ranged from as low as 10.7 mg kg^−1^ (Mrima Hill deposit, Kenya) to as high as 631.6 mg kg^−1^ (Matongo deposit, Burundi), while the concentrations in phosphate fertilizers ranged from 107.9 for an imported fertilizer to 281.0 mg kg^−1^ for a local fertilizer produced from Minjingu phosphate rock in Tanzania. In this context, it is noteworthy that the naturally occurring concentration of uranium in the earth crust is between 1.4 and 2.7 mg kg^−1^ and uranium mines in Namibia commercially process ores with uranium concentrations as low as 100–400 mg kg^−1^. This study thus confirms that East African phosphate rock, and as a result the phosphate fertilizer produced from it can contain relatively high uranium concentrations. Options to recover this uranium are discussed, and it is recommended that public–private partnerships are established that could develop economically competitive technologies to recover uranium during phosphate rock processing at the deposits with the highest uranium concentrations.

## Introduction

Phosphate rock and phosphate fertilizer are sources of elemental phosphorus which increases both soil fertility and crop yields (Mogollón et al. 2018; Hellal et al. 2019). Phosphate rock, pre-concentrated phosphate ore, can contain elevated concentrations of naturally occurring uranium that Bunus (2000) estimated to largely be in the order of 80–100 mg kg^−1^ for sedimentary ore with higher-end concentrations occasionally reaching 160–180 mg kg^−1^. The majority of this uranium (> 80%) transfers to the phosphate fertilizer stream during wet phosphoric acid production, the predominant process (> 90% of all fertilizer plants globally) to obtain mineral fertilizers from phosphate rock (Haneklaus et al. 2017a). The concentration of uranium in phosphate rock and resulting mineral fertilizers thus largely depends on the type of the phosphate ore used as a raw material. Phosphate rock can also be directly applied to agricultural soils without processing it into chemical fertilizer. Highly reactive phosphate rock that is agronomically suitable for direct application is for instance applied on more acidic soils, such as the ones found in East Africa (Casanova 1995; Rajan et al. 1996; Szilas 2002).

In the case of direct application of phosphate rock, all uranium contained in the phosphate rock is transferred to the agricultural soil. Removing uranium during phosphate fertilizer production, though commonly done on industrial scale in Florida in the USA in the 1980s–1990s (Steiner et al. 2020), is currently not preferred by fertilizer producers as it implies increased manufacturing costs. Removing uranium from phosphate rock destined for direct application is even more challenging since the heavy metal cannot be recovered from a liquid solution (as is the case during wet phosphoric acid processing) but would require a direct leaching approach (or similar) prior to the usual processing steps that would then be conducted with the remaining rock lattice. Al Khaledi et al. (2019) and Guzmán et al. (1995) previously proposed such an approach that is presently not considered economically feasible (Gabriel et al. 2013).

Essentially, during phosphate fertilizer production, uranium is presently neither recovered as a mineral resource nor removed as a contaminant because many countries do not have legislation and regulations in place that restrict uranium concentrations in mineral fertilizers (Kratz et al. 2016; Haneklaus et al. 2017b), and this practice is presently not economically viable (López et al. 2019; Haneklaus 2021; Shang et al. 2021). It is noteworthy though that since the cost for mining (especially a large part of the up-front capital costs as well as infrastructure development) are already taken care of by the phosphate industry (Reitsma et al. 2018), additional (byproduct) uranium recovery is currently at the edge of being monetarily profitable (Haneklaus et al. 2017b).

It is suspected though, and part of an active scientific debate, that prolonged application of phosphate rock and phosphate fertilizer can cause uranium accumulation on agricultural soils (Takeda et al. 2006; Yamaguchi et al. 2009; Schipper et al. 2011; Bigalke et al. 2017; Ratnikov et al. 2020; Campos et al. 2021). It is further speculated that a high concentration of uranium in agricultural soil could influence its uptake by plants through the root system in a similar way that other essential elements such as nitrogen, phosphorous, potassium, calcium, and magnesium and other supplemented micronutrients are absorbed (Velasco et al. 2009; Shtangeeva 2010; Sheppard 2011; Baumann et al. 2014; Harguindeguy et al. 2019; Semioshkina and Voigt 2021). Saleh et al. (2018) report for instance that the uptake of uranium from soils by plants behaves chemically similar to that of Ca. The concentration of uranium in phosphate rock and phosphate fertilizer is thus of concern and needs to be better understood.

Systematic and timely data on the uranium content of the most common phosphate fertilizers used across East Africa is not available today. This study was thus conducted to better understand the concentrations of uranium in phosphate rocks and phosphate fertilizers disseminated on agricultural soils in East Africa.

## Materials and methods

### The study area

**Fig. 1:**
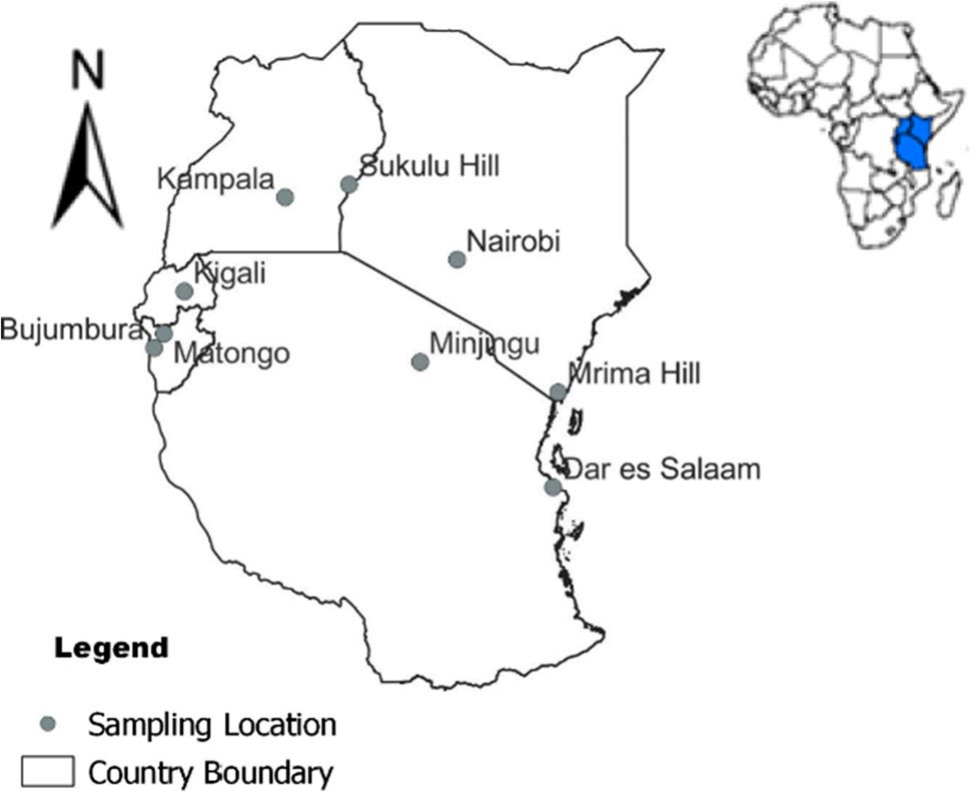
Phosphate rock and mineral fertilizer sampling sites of this study

Uranium concentrations from four major phosphate rocks and four commonly used phosphate fertilizer types in East Africa were determined. The study involved phosphate rocks collected from the following deposits: Matongo (Burundi), Minjingu (Tanzania), Mrima Hill (Kenya), and Sukulu Hill (Uganda). In addition, commonly used phosphate fertilizers were collected from traders in Arusha and Dar es Salaam (Tanzania), Bujumbura (Burundi), Kampala (Uganda), Kigali (Rwanda), and Nairobi (Kenya). The locations of the sample sides are depicted in Fig. 1.

### Sample collection and preparation

The phosphate rocks were collected from five randomly selected sampling points at about 2 m depth measured from the surface for each phosphate deposit with the aim of getting representative samples and minimizing potential influences from weathering or vegetation. The five samples were placed in a clean polythene sheet, and a composite sample of about 1 kg mass was drawn and carried to the laboratory for further processing. In the laboratory, the samples were crushed, ground, homogenized, and sieved using a 60-μm diameter sieve. Three replicates were drawn, packed, and labeled in clean airtight zip-lock polythene bags for further laboratory processing.

The phosphate fertilizer samples were collected from fertilizer dealers in each country. In addition, 5 kg of phosphate fertilizers were collected from the capital city of each country (a total of 25 samples). The collected phosphate fertilizers from each sampling site were again combined to get four representative samples.

Two fertilizers are locally produced in northern Tanzania and used in East Africa. These are Minjingu organic hyper phosphate (MOHP) with the following sale specifications: P_2_O_5_: 28%, MgO: 2.5%, CaO: 36%, and Minjingu Nafaka Plus (NPS) with the following sale specifications: N: 9%, P_2_O_5_: 16%, K_2_O: 6%, CaO: 25%, S: 5%, MgO: 2%, Zn: 0.5%, and B: 0.1%. Diammonium phosphate (DAP) (18:46:00) and nitrogen phosphorus and potassium (NPK) (23:23:00) are also locally used but are mostly imported from Morocco and Saudi Arabia. Samples of these imported fertilizers were also analyzed.

The phosphate rocks and phosphate fertilizer samples were oven-dried at 100 °C to remove moisture to a constant weight. The phosphate rocks were then crushed, milled, and sieved using a 60-µm sieve size, while the phosphate fertilizers were milled using a RETSCH Cross Beater mill machine and sieved using a 63-µm sieve. An aliquot of 4 g was subsampled for energy-dispersive X-ray fluorescence (EDXRF) measurements. The subsample was processed by mixing with a 0.9-g clean wax binder (FluXANA CEREOX®, Germany). The mixture was poured into an 80-mL polytetrafluoroethylene grinding bowl with 3 agate grinding balls of 20-mm diameter each, inserted into a Pulverisette 6 planetary mono mill® (Fritsch GmbH, Germany), and set to 150 rpm for 120 s to achieve a fine milling powder that can then be used in the subsequent experiments (Mwalongo and Mohammed 2013). The homogenized mixture was poured into a cylindrical pressing die with an inside diameter of 32 mm. The mixture was pressed using a manual hydraulic press, and tablet-like pellets were formed for further analysis.

### Uranium measurement using EDXRF

The EDXRF measurement system was calibrated using multi-elemental standard reference material from the National Institute of Science and Technology (NIST 2711a—Trace Metals in Soil) and re-confirmed by using International Atomic Energy Agency (IAEA) certified reference materials (IAEA 312 and 314) of a similar matrix. The detection limit for the EDXRF technique for elemental uranium was 1.41 ± 0.07 mg kg^−1^.

The validation process aimed at confirming the fit for purpose in measuring uranium concentrations in unknown field samples. The result is shown in Table 1. The ratio of the laboratory-measured value to the certified value ranged from 0.968 to 1.032 and was thus within ± 3%.

**Table 1:** Measurements of the standard reference materials

Reference material	Uranium concentration (mg kg^−1^)		
	Certified value	Measured value	Ratio
NIST 2711A	3.10	3.20	1.032
IAEA-312	16.5	15.97	0.968
IAEA-314	56.8	55.7	0.981

### Statistical analyses

The statistical data analysis was performed using STATISTICA 8th Edition software (StatSoft, Inc., Tulsa, OK, USA). Uranium concentrations were analyzed based on the interactions among the phosphate rocks and phosphate fertilizers and each factor individually. The two-way ANOVA statistical analyses were performed with treatments being phosphate rock sources as the main factor and phosphate fertilizer type as a subfactor. For the isolation of interaction and individual effects of sites (East African countries), phosphate rock, and phosphate fertilizers, a post hoc Tukey’s honestly significant difference multiple comparison test was used due to a higher degree of freedom (five countries × four fertilizer types = 20 for the measured variables). The significance threshold was set at *P* = 0.05 and *P* = 0.001 for high significance. The treatment means were compared by the standard error of the mean difference.

## Results and discussions

### Concentrations of uranium detected in major phosphate rocks in East Africa

The concentration of uranium measured in phosphate rocks from East Africa is shown in Table 2. The results of the uranium measurements vary significantly among the different countries and are generally (except for the samples from Mrima Hill, Kenya) high to very high if compared to uranium concentrations at phosphate rock deposits around the world reported by Haneklaus (2021). The highest uranium concentration of 631.6 ± 2.5 mg kg^−1^ was recorded for the Matongo phosphate rock deposit (Burundi), and the lowest uranium concentration of 10.7 ± 0.2 mg kg^−1^ was recorded for the Mrima Hill phosphate rock deposit (Kenya). The Minjingu phosphate rock deposit (Tanzania) showed a uranium concentration of 446.1 ± 0.4 mg kg^−1^, and the Sukulu Hill deposit (Uganda) showed a uranium concentration of 120.6 ± 0.3 mg kg^−1^. It is noteworthy that the naturally occurring concentration of uranium in the earth crust is estimated to be between 1.4 and 2.7 mg kg^−1^ (Haynes et al. 2016; WNA 2021) and uranium mines in Namibia on the other side of the continent commercially process ores with uranium concentrations as low as 100–400 mg kg^−1^ (WNA 2022).

**Table 2:** Uranium concentration in major phosphate rock deposits in East Africa

Name of the phosphate rock deposit	Deposit type	Country	Uranium (mg kg^−1^)
Matongo	Igneous	Burundi	631.6 ± 2.5
Minjingu	Sedimentary	Tanzania	446.1 ± 0.4
Sukulu Hill	Igneous	Uganda	120.6 ± 0.3
Mrima Hill	Igneous	Kenya	10.7 ± 0.2

Since phosphorus is the primary element for which phosphate rock is mined, the quality of the phosphate rock is usually classified by its P_2_O_5_ concentration. Phosphate rock with a P_2_O_5_ concentration of 12–16% is considered lower grade and 17–25% medium grade, and in high-grade ores, the P_2_O_5_ concentration is above 26% (Boujlel et al. 2019). Based on this classification, the analyzed samples from the Sukulu hill and the Minjingu deposit can be considered high-grade phosphate rocks, while the samples from the Matongo deposit can be considered a medium-grade phosphate rock and samples from the Mrima hill deposit a low-grade phosphate rock (Table 3).

**Table 3:** Chemical composition of major phosphate rocks and common phosphate fertilizers in East Africa

	P_2_O_5_ (%)	K_2_O (%)	CaO (%)	MgO (%)	CaO/P_2_O_5_
Name of the phosphate rock deposit					
Sukulu Hill	30.57 ± 0.07	0.32 ± 0.01	39.56 ± 1.30	0.63 ± 0.08	1.29
Minjingu	34.23 ± 0.30	1.95 ± 0.01	51.81 ± 0.41	4.58 ± 0.35	1.51
Matongo	17.65 ± 0.26	1.60 ± 0.03	13.02 ± 0.30	0.34 ± 0.08	0.74
Mrima hill	3.5 ± 0.01	ND	0.61 ± 0.02	9.39 ± 0.20	0.17
Name of the phosphate fertilizer					
DAP	34.61 ± 2.91	ND	25.89 ± 2.95	1.75 ± 0.25	0.74
MOHP	26.47 ± 1.19	ND	25.75 ± 2.40	1.84 ± 0.03	1.39
NPS	14.83 ± 0.25	4.41 ± 0.33	34.63 ± 2.50	1.73 ± 0.16	2.34
NPK	21.12 ± 0.32	ND	24.32 ± 1.57	1.74 ± 0.32	1.61

*ND* not detected

The relatively high uranium concentration at the Matongo phosphate rock deposit can be attributed to the syenite complex formation that contains thorium-uranium-potassium anomalies. It was earlier found that the deposit has high impurities that do not support using the raw material for the production of superphosphate fertilizer (Van Straaten 2002). This can be expressed through the CaO to P_2_O_5_ ratio which is 0.17. In this study, Matongo phosphate rock was found to contain a P_2_O_5_ content of 17.65%, which is higher than the 0–15% and 11–13% P_2_O_5_ content previously reported by Songore (1991) and Van den Berghe (1996). The difference may be attributed to different sampling strategies and variations of P_2_O_5_ concentration within the deposit. Furthermore, Matongo phosphate rock has the lowest MgO (0.34 ± 0.08%) but a substantial K_2_O (1.60 ± 0.03%) content. Matongo phosphate ore is a low-grade phosphate ore whose development could nonetheless be interesting if not only its P_2_O_5_ content, but the other valuable materials are considered for recovery.

The Minjingu phosphate rock deposit also contained elevated concentrations of uranium that are usually attributed to the ores high organic matter content (Szilas 2002). The Minjingu phosphate rock deposit is a layered phosphate deposit comprising of remaining organic matter and dead animals sedimented in a paleo-rift valley environment (Schlüter 1997). Our study also observed that Minjingu phosphate rock has a high P_2_O_5_ content (> 30%) and a relatively high CaO to P_2_O_5_ ratio of 1.51. The Minjingu phosphate rock deposit had a MgO concentration of 4.58 ± 0.04% and a K_2_O concentration reaching 1.95 ± 0.01%.

The Sukulu Hill deposit had an average uranium concentration of 120.6 mg kg^−1^ and the second highest P_2_O_5_ concentration of 30.57%. It is an alkaline igneous carbonatite phosphate rock deposit used to produce phosphate fertilizer in Uganda through the wet phosphoric acid process. The produced fertilizer is mostly used in Uganda (Kisitu 1991; Nakasango 2021) and not exported. The phosphate rock has a relatively low MgO content and CaO/P_2_O_5_ ratio making wet phosphoric acid processing possible. In addition, the Sukulu Hill phosphate rock deposit has low reactivity that could be attributed to a relatively high iron oxide content, so that the material is not feasible for direct application (Butegwa et al. 1995).

Samples from the Mrima Hill deposit had the lowest uranium concentrations (10.7 ± 0.2 mg kg^−1^) and a very low P_2_O_5_ content of 3.5 ± 0.2 mg kg^−1^ which raises the question if they should be considered a phosphate rock deposit at all. As a result of the low P_2_O_5_ content, the deposit is presently not mined. It might eventually be developed for its MgO content (9.39 ± 0.20%) rather than the traces of P_2_O_5_.

The selected macronutrient content (P_2_O_5_ and K_2_O and MgO, CaO) of the common phosphate fertilizers used in East Africa were assessed and are shown in Table 3. Appropriate supply of nutrients is one of the important factors to assess the quality of the fertilizer supplied to farmers for meeting soil requirements and improving yield.

Assessing the content of macronutrients such as P and K expressed as P_2_O_5_ and K_2_O respectively are essential. Depending on soil conditions, crop types, and other agronomical factors, the fertilizers usually include other secondary macronutrient oxides in the form of CaO and MgO. During manufacturing, fertilizers are produced by either using compound processes where NPK are homogeneously mixed in one granule or bulk blending where the nutrients are in separate granules (Morari et al. 2011).

The average macronutrients for the four common phosphate fertilizers (DAP: 18:46:00; MOHP: P_2_O_5_: 28%, MgO: 2.5%, CaO: 36%; NPS: 9:16:06, and NPK: 23:23:00) used in East Africa was assessed. The measured results were compared with the manufacturer’s declared value on the label. In DAP, the average P_2_O_5_ was 34.61 ± 2.91% compared to 36% provided by the manufacturer. The K_2_O was not detected in DAP samples (not present in fertilizer formulation), and the CaO average concentration was 25.89 ± 2.95%.

The average P_2_O_5_ concentration for MOHP was 26.47 ± 1.19% compared to 28% specified by the manufacturer. This difference is within the tolerable limit of 1.1% set by the Kenya Bureau of Standards (KeBS 2018). The measured MOHP CaO concentration (25.75%) was compared with the 36% quoted by the manufacturer. Our assessment suggests that it was overdeclared by about 28.4%, and the concentration of MgO was 1.84 ± 0.03% compared to the manufacturer’s quoted value that was 2.5%. This first analysis indicates that the macronutrient may be overstated by as much as 26%, but more systematic studies would be needed to get a clearer picture.

The NPS fertilizer average P_2_O_5_ measured was 14.83 ± 0.25% compared with 16% stated by the manufacturer. The result is within the tolerable limits specified by East African Authorities. The average concentration of MgO and CaO were 1.73 ± 0.16% and 34.63 ± 1.6%, respectively. Our results are in agreement with data published by Szilas (2002) who observed that the MgO and CaO content varied from 0.17 to 4.05% and 28.91 to 50.72%, respectively. The K_2_O was 4.4% compared with the manufacturer’s quoted value (6%); Szilas (2002) reported the K_2_O to range from 0.1 to 2.59%, which implies that the NPS K_2_O was overdeclared by 26.7%. The NPK average P_2_O_5_ was 21.12 ± 0.32% compared with the manufacturer’s quoted value of 23%, which was within the recommended tolerable standards.

The average concentration for macronutrient oxides, P_2_O_5_, and K_2_O in all phosphate fertilizers ranged from 14.83 ± 0.25% to 34.61 ± 2.91%. The macronutrients oxide MgO and CaO ranged from 1.73 ± 0.16% and 1.84 ± 0.03%. These results indicate that fertilizer manufacturers’ declared nutrients did not ascertain 100% matching with the fertilizer formulation shown on the manufactured fertilizer labels. Some nutrient formulations were within or not within the tolerance specification given by the East African fertilizer standards regulatory bodies. Overall, the reported major nutrients complied with the East African countries’ fertilizer standards. The calculated ratio of CaO/P_2_O_5_ ranged from 0.74 to 1.6, which is within the acceptable fertilizer value in agriculture (Kawatra and Carlson 2013). Although the phosphate fertilizers manufactured from Minjingu phosphate rock have higher uranium concentrations, the quality of the fertilizers is still good and meets the set standard.

### Uranium concentration in major phosphate fertilizers used in East Africa

The measured uranium concentrations in the phosphate fertilizers are shown in Table 4. Tanzania had 226.48 ± 13.81 mg kg^−1^ the highest uranium concentration in this study followed by Kenya with 187.07 ± 11.64 mg kg^−1^, Rwanda with 174.71 ± 16.72 mg kg^−1^, Uganda with 152.63 ± 11.58 mg kg^−1^, and Burundi with 136.37 ± 11.67 mg kg^−1^.

**Table 4:** Average uranium concentrations of common phosphate fertilizers used in East Africa

Country	Uranium (mg kg^−1^)
Burundi	136.37 ± 11.67
Kenya	187.07 ± 11.64
Rwanda	174.71 ± 16.72
Tanzania	226.48 ± 13.81
Uganda	152.63 ± 11.58
Type of fertilizer	
DAP	107.88 ± 9.60
NPS	203.57 ± 18.40
MOHP	281.57 ± 15.82
NPK	108.79 ± 29.00
2-way ANOVA F-statistics	
Countries (C)	189.72*
Fertilizers (F)	1391.03*
C × F	285.36*

The values in the table are mean ± SE (standard error), and * is significant at *P* ≤ 0.001

**Fig. 2:**
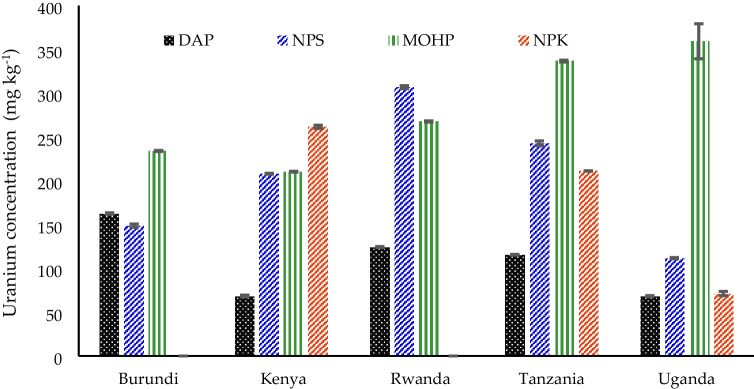
Uranium concentration in common phosphate fertilizers used in East Africa

The uranium concentration reported for Tanzania can largely be attributed to the common use of Minjingu phosphate rock as a raw material in fertilizer production (Makweba and Holm 1993; Banzi et al. 2000; Meza et al. 2015) or even in direct application after simple beneficiation (Mnkeni et al. 1991; Kifuko et al. 2007; Szilas et al. 2008; Kalala and Semoka 2010). MOHP and Nafaka Plus, a NPS fertilizer produced from Minjingu phosphate rock, both have elevated uranium concentrations (see Table 4 and Fig. 2). These fertilizer products are used on acidic soils and are also exported to neighboring countries. Kenya uses considerable amounts of fertilizer products derived from Minjingu phosphate rock (Kifuko et al. 2007; Ndungu-Magiroi et al. 2015; Ndeleko-Barasa et al. 2021) and thus has the second highest average uranium concentration among the investigated countries in this study as indicated in Table 4.

The imported NPK and DAP fertilizers to East African Countries recorded the lowest uranium concentrations of 107.9 ± 9.6 mg kg^−1^ and 108.8 ± 29.0 mg kg^−1^, respectively. These levels are almost similar to other NPK and DAPs from Western and Northern Africa (Yamazaki and Geraldo 2003). NPK concentrations for Kenya in Fig. 2 are relatively high since the country imports phosphate ore from Minjingu with elevated uranium content to produce NPK fertilizer.

Detailed uranium concentrations in the different fertilizers by country are provided in Fig. 2. A considerable variance in the uranium concentration in the same fertilizer types could be observed that may be attributed to different national fertilizer nutrient requirements. MOHP fertilizer showed the highest uranium concentrations with 336.6 ± mg kg^−1^ detected in fertilizer obtained in Tanzania, 359. 2 mg kg^−1^ in Uganda, 267. 67 mg kg^−1^ in Rwanda, 234.0 mg kg^−1^ in Burundi, and 210.4 mg kg^−1^ in Kenya. Similarly high differences were observed for NPS fertilizer in Rwanda (306.8 mg kg^−1^), Tanzania (242.6 mg kg^−1^), Kenya (208.0 mg kg^−1^), Burundi (148.8 mg kg^−1^) and Uganda (111.6 mg kg^−1^).

The NPK and DAP fertilizers used in East African countries are directly imported from China, Egypt, Morocco, and Saudi Arabia (UN Comtrade 2022). Phosphate rocks in these countries show lower average uranium concentrations than the analyzed phosphate rocks from East Africa. Average uranium concentrations are approximately 27 mg kg^−1^ for China (though higher concentrations have been measured by Ye et al. (2019)), 90 mg kg^−1^ for Egypt, 97 mg kg^−1^ for Morocco, and 100 mg kg^−1^ for Saudi Arabia (Khater 2012; Tulsidas et al. 2019; Haneklaus 2021) so that the resulting fertilizers show lower uranium concentrations than the fertilizers produced from local phosphate rock with higher uranium content in East Africa. A recent study from Ramteke et al. (2022) on the uranium content of imported mineral fertilizers marketed in India that are from similar sources than the imported once sold in East Africa is in good agreement with this work.

The frequent use of MOHP and NPS could have resulted in the accumulation of uranium in East African soils, and further systematic studies as they were for instance reported by Sun et al. (2020a, b; 2022) are recommended. First studies by Mlwilo et al. (2007) as well as Nkuba and Mohammed (2014) already observed radioactivity above background levels in common crops such as maize and mung beans. Here again systematic and recent studies that consider environmental risk assessments for the local population are highly recommended. As a result of the relatively high uranium concentrations found in phosphate rocks in East Africa, it is further recommended to establish public–private partnerships that can investigate the techno-economic feasibility of commercial uranium recovery during fertilizer production.

## Conclusions

This study investigated uranium concentration in major phosphate rocks of East African origin and phosphate fertilizers used in Burundi, Kenya, Rwanda, Tanzania and Uganda. Besides, major elements from the phosphate rocks of East Africa were also assessed. It is evident from this study that East African phosphate rocks particularly those from Matongo (Burundi) and Minjingu (Tanzania) contain elevated concentrations of uranium (636.6 mg kg^−1^ and 446.1 mg kg^−1^, respectively) if compared to phosphate rocks mined globally that are usually in the range of 80–120 mg kg^−1^ with higher-end concentrations of 160–180 mg kg^−1^ (both for sedimentary ores) reported in the literature. Not surprisingly, this study could subsequently show that mineral fertilizers produced from East African phosphate rocks also contain higher concentrations of uranium than fertilizers produced from raw material with lower uranium concentrations. Uranium can be recovered during phosphate rock processing, and we recommend additional studies/risk assessments to better understand possible accumulation of radionuclides on soils and in plants in East Africa as well as the establishment of public–private partnerships that could develop specific economically competitive technologies to recover uranium during phosphate rock processing at the deposits with the highest uranium concentrations.

## Data Availability

All data used that was not obtained through own measurements by the authors is openly accessible.
